# Exploring the Perceived Risks and Benefits of Heroin Use among Young People (18–24 Years) in Mauritius: Economic Insights from an Exploratory Qualitative Study

**DOI:** 10.3390/ijerph17176126

**Published:** 2020-08-23

**Authors:** Gareth White, Susan E. Luczak, Bernard Mundia, Smita Goorah

**Affiliations:** 1 Health Policy and Financing Unit, Department of Public Health, Institute of Tropical Medicine Antwerp, 2000 Antwerp, Belgium; 2 Department of Psychology, Dornsife College of Letters, Arts, and Science, University of Southern California, Los Angeles, CA 90089-1061, USA; luczak@usc.edu; 3 Kenya AIDS NGO Consortium, Regent Court, P.O Box 69866-00400 Nairobi, Kenya; benmundiaasmara@gmail.com; 4 Department of Medicine, Faculty of Science, University of Mauritius, Réduit 80837, Mauritius; sm.goorah@uom.ac.mu

**Keywords:** risk perception, heroin use, young people, harm reduction, health economics, Mauritius, HIV/AIDS

## Abstract

The decreasing age of young people injecting illicit drugs is an under-reported challenge for the prevention of HIV transmission worldwide. Young people aged 15–24 years represent 1 in 5 persons living with HIV in Mauritius where the epidemic is driven by injecting drug use and risky sexual behaviours. We recruited 22 heroin users aged 18–24 and 5 service providers working in harm reduction (HR) for the present study. Qualitative data were collected through unstructured interviews. We adopted an economic framework and an inductive approach to the analysis, which implied revising codes and themes. The risks heroin users described as consumers of illicit drugs and as clients of HR services could not be analyzed in isolation. Polydrug use emerged as a recurrent coping mechanism resulting from the changing dynamics within the heroin market. The risks faced by women went beyond addiction and infection with HIV. How participants viewed the risks and benefits linked to using heroin was greatly influenced by gaps in knowledge that left room for uncertainty and reinforcing mechanisms such as peer influence. The study shows that qualitative research can produce in-depth socio-behavioural insights required to produce more effective services for young people.

## 1. Introduction

The decreasing age of initiation into injecting drug use suggests a significant change in the age composition of the global population of people who inject drugs (PWIDs). This can potentially exacerbate the complex relationship between HIV transmission and injecting drug use. Injecting drugs at a younger age does not imply accessing the services required to mitigate the health, psychosocial, and legal impact of drugs. Moreover, individual drug history differs greatly as illicit drug use exists across social strata and is a multifaceted issue linked to different factors. Variations also exist in the initiation into long-term injecting drug use, further highlighting the changing nature of risks experienced by young users. These issues are under-researched. Thus, exploring the relationship between young heroin users and a small black market for illicit substances within an island state can help to identify fundamental dynamics and ways to reduce risks.

### 1.1. Context

The Republic of Mauritius is a high middle-income economy found in the Indian Ocean which has reached an advanced stage in its epidemiological transition with a life expectancy of 74.8 years and a population of 1.22 million [1]. Most deaths are attributed to noncommunicable diseases (69.2%) yet, preventing and mitigating the burden of infectious diseases such as human immunodeficiency virus (HIV) and hepatitis remains a priority. Since the first case was detected in 1987, the HIV epidemic has been driven mainly by injecting drug use and sexual intercourse among key affected populations [2].

Heroin in a form known as “Brown Sugar” has been available in Mauritius since the late 1970s [3]. In more recent times, illicit substances such as heroin, ecstasy, and Black Mamba were perceived as the most dangerous substances on the market [4]. The most popular substances include marijuana, followed by heroin and “subutex” (also known as buprenorphine, which can also be injected), “Lablanche” (or “white heroin”), and tranquilizers [5]. In 2010, the price of 1g of heroin in Mauritius was one of the highest in the world and was estimated at USD 329.35 [6]. Other countries where heroin was more expensive (sometimes depending on the type, at that time) included Brunei, New Zealand, Georgia, Lao People’s Democratic Republic and Cambodia. There has been no official information on the quality or purity of the heroin available on the streets of Mauritius since 2003 when it was estimated at 20.6% [7]. Progressive measures known as harm reduction (HR) were introduced in 2006 to mitigate the growing impact of HIV and related conditions [8]. In the Mauritian context HR encompasses the provision of health services to illicit drug users such as needles and syringes as well as methadone substitution therapy delivered without judgment. The overall aim of such services is to prevent the incidence of HIV and hepatitis B and C (“HBV” and “HCV”, respectively) and minimize social and legal impacts associated with illicit drug use.

Key affected populations include PWIDs, sex workers (male, female and transgender) and men who have sex with men (MSM). HIV prevalence is currently estimated at 32.3% among PWIDs, 28.4% among transgender individuals, 17.3% among prisoners, 17% among MSM, and 15% among sex workers (female) [9]. Additionally, 88.8% of the PWIDs and 95.8% of those infected with HIV are co-infected with HCV. The total number of individuals in the 15–24 age group detected since the beginning of the epidemic in 1987 is 1274 [10]. A recent increase in the number of clients of needle and syringe programmes (NSP), suggests a growing trend in injecting drug use [11,12]. While there have been some instances of young children infected through prevention of mother-to-child transmission (PMTCT), it is highly likely that most of the 698 HIV cases officially detected among those aged 15 to 24 years between 2008 and 2017 are outcomes of risky injecting practices and sexual behaviours which exist despite joint interventions by both non-state actors and the government.

### 1.2. The Economics of Risky Behaviours

From a traditional economic perspective, the behaviour of individuals emanates from decisions which involve weighing the costs and benefits of a given situation. Ideally, this individual process is informed by existing preferences, always resulting in optimal decisions and are shaped by rational choice. The aggregate of such behaviours results in what is referred to as a market where individuals make choices according to a combination of means, preferences and other alternatives referred to as “consumer choice theory”. The introduction of psychology within economics has allowed the discipline to evolve both in terms of assumptions and methods used. For instance, “prospect theory”, which was influenced by psychology and paved the way for behavioural economics as a sub-discipline, recognises that decisions are not always optimal [13]. Hence, the willingness to take risks is influenced by framing options in terms of perceived risks/losses and rewards/gains. Decisions are context-dependent and subjective. Merging psychological models of decision making with economic frameworks can produce useful paradigms to investigate addiction and individual substitution behaviours, compliance with treatment, self-control as well as black market responses to drug control [14]. From this perspective, heroin use presents risks but also perceived ‘gains’ or ‘benefits’. Using qualitative methods represents an opportunity to examine how well traditional economic theories apply when confronted with real-world events [15].

Risk perceptions, here defined as how individuals perceive a threat by interpreting and weighing consequences against benefits, became a recurring aspect of the present study upon hearing the choices, behaviours, and preferences expressed by young people who use heroin. Often associated with positive behaviour change in healthcare, exploring perceived risks can be a starting point to understanding behaviour by viewing individuals as everyday decision makers for their own health. According to the tripartite model for risk perception [16] there are three distinct forms according to which health risks are perceived. These consist of *(1) deliberative:* risk perceptions which are systematic, logical, and rule-based; *(2) affective:* risk perceptions which are driven by emotions but can be optimal for judgment and decision-making in given circumstances; *(3) experiential:* which may combine both and are based on experience.

**Figure 1:**
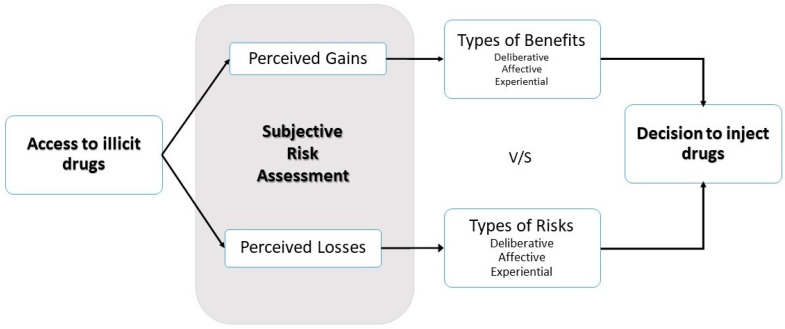
Weighting the perceived gains vs. losses of injecting drug use: a behavioural economic framework.

Risk and behaviour have been researched and discussed extensively in relation to drug use and HIV. The discussion has evolved from discussions on the limits of individual rationality to a more socially organised phenomenon—among the already marginalized—to being a socially, culturally and politically determined condition [17,18]. While social forces and relationships are bound to impact on how risks are framed and re-framed by individuals, depending on their context and circumstances, an economic lens was adopted for the present study after listening to participants describe the benefits of injecting drug use. Such statements were reminiscent of behavioural economics and research carried out on the perceived quality of substances [19]. Such perceived benefits were not investigated in previous studies carried out in Mauritius. Benefits appeared to fall into the same three main categories as perceived risks and also include deliberative, affective, and experiential benefits. Adopting a perceived risks/benefits framework was deemed compatible with the overall aim of HR to minimize threats to users without passing judgment on the decision or compulsion to use illicit drugs. The initial framework developed after carrying out a few interviews is summarized in Figure 1.

### 1.3. Aims and Research Questions

Changes in drug consumption patterns suggest the emergence of new challenges. The overall aim of the study was to gain insights into the needs of young heroin users in Mauritius in order to design HR interventions which reduce the risk of HIV transmission and other emerging health-related problems linked to using illicit substances. Specific research questions included: (1) What is being consumed and what are the selection factors influencing the choices made by young people when buying? (2) How do young HR clients perceive the risks and benefits linked to regular heroin use?

## 2. Materials and Methods

The study is derived from a larger mixed method research titled: “Exploring Access and Utilisation of Needles & Syringes by Young People (18–24 Years) in Mauritius: An Inductive Rapid Assessment”. It was conducted between March 2017 to June 2018 on behalf of Collectif Urgence Toxida (CUT), which was the main NGO responsible for the distribution of needles and syringes at the time. It was part of a regional research exercise also conducted in Kenya and Tanzania. Face-to-face, unstructured interviews were conducted following the need for an in-depth exploration of individual client needs. Our team of interviewers developed topic guides for the study based on gaps found in the literature pertaining to the needs of heroin users who either smoked or injected. The team included an anthropologist working for an NGO involved in HIV/AIDS prevention and advocacy, a clinical psychologist working in social development, and the principal investigator, a health economist involved in health systems strengthening. The guide was discussed with CUT fieldworkers. We carried out a pilot study with three clients of CUT and one service provider to refine our approach to data collection. Ethical clearance to carry out this study was obtained from the Ministry of Health and Wellness (previously known as the Ministry of Health and Quality of Life) in August 2017. It was agreed that the ministry would receive progress reports until preliminary findings were available. We shared such findings with the ministry in March 2018. Both the design of the qualitative study and its expected outcomes were discussed with stakeholders from different organisations during a consultative workshop in September 2017. This helped us to increase collaboration across institutions and refine the recruitment strategy of participants.

### 2.1. Sampling and Recruitment Procedure

The inductive nature of this study combined elements from different forms of qualitative research which include phenomenology and grounded theory. These approaches are reflected in the design of the sample. We used both snowballing and purposive sampling following challenges in recruiting young heroin users who are seen as a hidden population in Mauritius. Fieldworkers from various NGOs screened potential participants within the agreed target group before we started to interview. We encouraged participants to refer their peers based on their experience. All fieldworkers signed a contract which explained the terms and conditions of their employment and resulting remuneration. The peer recruitment strategy was successful. We were able to interview 22 individuals, aged 18–24: 11 men and 11 women aged 18–24, who had injected drugs within the past 4 months and 5 service providers working in HR. We conducted interviews outside well-known clusters of users (e.g., in districts such as Plaines Wilhems and Port Louis) to obtain diverse perspectives from different types of clients. We asked service providers (Institutions and individuals providing HR services) to participate in the study following: (1) their willingness to contribute to the consultative workshop organised in September 2017 and (2) existing experience in the field. Participants and selected service providers (a volunteer and a ‘hit doctor’: an informal title given to an experienced injecting drug user who provides guidance to young users), were given a voucher for groceries, food or clothes at the end of the interview as compensation for their participation. 

Different schools of thought provide different recommendations on how to achieve saturation of subjective experiences and perceptions. The recommended sample sizes for phenomenological studies is 5 to 25 participants and a maximum of 30 for grounded theory [20]. Hence, we conducted a total of 27 interviews until saturation of themes occurred, as per the ultimate aim of grounded theory [21].

### 2.2. Data Collection

Our team designed the initial topic guides and performed the interviews. Fieldworkers who had screened and recommended participants were asked to stay nearby but did not participate in the interview. Participants were given a consent form to sign prior to the interview. This document provided an outline of the study and guaranteed both anonymity and confidentiality before, during, and after the interview. Interviewers provided an overview of both the study and the interview process before starting. We also informed participants of their right to stop the interview and leave at any time without any repercussion. Participants were also made aware that findings from the study would be disseminated through a report, academic publications, strategic communications, and workshops. 

Our team favoured a more unstructured approach to the interviews after the pilot stage, once we tested the questions from the topic guide and reached a deeper understanding of the context. Thereon, we gradually encouraged participants to express themselves in their own terms. Key themes which we discussed with them included drug history and use, perceived risks linked to drug use and access to HR services. Our clinical psychologist acted as a mediator and helped to manage unease and negative reactions when participants discussed their personal lives. Some additional interviews were carried out with individuals deemed to be stable by fieldworkers and who did not require the presence of a clinical psychologist. Interviews were carried out and recorded in Mauritian Creole from June 2017 to March 2018. The analysis team selected illustrative quotes based on the following criteria: a short quote was used if it was a recurrent theme whereas longer quotes were used if they provided insights into events, relationships, attitudes, or practices which were previously undocumented.

## 3. Results

This section describes the characteristics of participants and presents the main thematic findings of our study relating to the market for illicit drugs, perceived risks, and benefits of heroin use, as identified during the analysis.

### 3.1. Participant Characteristics

We interviewed hard to reach sub-groups such as sex workers (3 disclosed), former prison inmates (2) as well as methadone (1) and detoxification clients (2) through referrals from participants. Among the heroin users recruited, 11 participants were injecting heroin only, 7 were injecting heroin and using other substances. One of the participants believed she might be injecting amphetamines and using new psychoactive substances (NPS) while another participant had stopped injecting and was now only smoking heroin. Hence the term ‘heroin user’ is used instead of PWIDs as it describes both individuals who smoke heroin and those who inject. A participant was receiving codeine treatment for addiction but had injected heroin on a few occasions. Another participant had stopped injecting over the past month and was receiving methadone treatment but was taking ‘vert’, or ‘green’, an NPS on a regular basis. This was also the case of a participant who had stopped injecting over the past 3 months but was still smoking NPS. Being HIV positive was disclosed openly by three participants during their individual interviews. Service providers included: a volunteer who redistributed syringes but was not using drugs, a hit doctor, a peer educator who recently stopped using drugs, an NGO representative and a service provider from the public sector. The profiles of the participants interviewed for the present study are summarized in Table 1.

### 3.2. Local Market for Illicit Substances

According to the young heroin users and service providers in our study, the market for heroin was characterised by fluctuations in availability, price and quality. Illicit substances and heroin were relatively easy to find in both urban and rural areas. Such substances were even offered for free during the initial trial phase in some cases. The reasons for using included a mix of ease of access, developing higher tolerance, the need for a greater high, trauma, and curiosity. Other factors included price and peers. A participant who had recently been released from prison and had started injecting on the same day provided an overview of the current street price of drugs which was later complemented with our remaining interviews. Our findings are summarized in Table 2. 

Despite only coming into the limelight since 2015 (2 years prior to the study), it was mentioned in several interviews that NPS had been on the market for several years already. It was seen as a superior alternative to marijuana but also as much more addictive.

*“Marijuana’s high is a normal high. The first time I smoked synthetic, I felt different, the high was stronger. Sometimes you can’t control it. And sometimes when we do it, we don’t take it again for another week. And then one week we want to smoke it again. And then we smoke it again and then perhaps in another two days, we want to do it again. We smoke again and again until we get used to it. And then we do it every day.”*

*BV-P1*

#### Selection Factors

General external shocks to the overall drug market itself did impact on the market for heroin. Such trends encouraged some participants to adapt their consumption and behaviours accordingly, especially in case of shortage. For instance, a participant mentioned that the price of other drugs was a determining factor in deciding to inject. In this case, obtaining prescription drugs on the black market became more expensive than buying heroin, which was deemed as more accessible following individual means and implied a limited budget. Thus, heroin was seen as a better option and a good substitute.

*“I didn’t get high enough on weed. I didn’t feel right but when I took the syrup and the pills and all that, I felt better. But afterwards, the syrup and the pills, they became too expensive.”*

*BV-P5*

The consumption of injectable drugs was explained in terms of preferences and availability of substances used as complements to heroin such as Rophynol, or opioid substitutes such as buprenorphine. Availability and variance in price were mentioned by a participant. The price of heroin did fluctuate according to the location and was likely to impact on the amount consumed according to a service provider. The quality of the substance available was a recurrent theme noted when assessing the characteristics of the heroin market. Several participants complained that, in general, the quality of the heroin available on the streets had been inferior for several years, although with some geographic differences.

*“Well, maybe some three or four years since it has changed, it’s like you don’t… sometimes you get… it’s like now, sometimes you get the good quality, sometimes you get the bad quality. Most of the time, it’s the bad quality.”*

*F-P1*

A possible explanation provided by a service provider on this issue was the very lengthy ‘time to market’ of heroin due to a long supply chain from production to distribution. During that interval, the substance is usually diluted several times so that by the time it reaches Mauritius, where it is further diluted, its potency is already much lower. As a result of the lower quality, consuming heroin became more expensive to obtain the same high, following long-term use. Yet, the price usually remained the same, irrespective of quality. Heroin users were likely to feel that *“you have to buy more … to not get cravings”* or to adapt new consumption patterns. Dealers could also sell substitutes like buprenorphine to compensate for the quality of heroin since *“They make more money with it than with brown.”* Available at the same price as heroin, the pleasure or ‘high’ derived from buprenorphine was described by a participant as being better and as lasting for longer (2 days) in some cases when injected. However, withdrawal from buprenorphine was also described as being harder. Methadone, another substitute for opioids, was also consumed illegally in some cases, or combined with other drugs, such as NPS, by registered patients. Coping behaviours related to poor quality heroin among young heroin users also included mixing different forms of heroin, obtained from different sources *“Yes, it doesn’t matter even if it came from two different sources, as long as it’s brown.”* Another approach was to mix heroin with different drugs, referred to as ‘polydrug use’ among heroin users.

*“Well when I first started, we used to share one dose between two of us. And then with time, I started feeling that one dosage between two people wasn’t enough because I couldn’t feel anything. It was like I was normal after I’d taken it. Then we’d take two doses for two people. And then with time, we started taking an eighth (of 1g). Then even that wasn’t enough. Then I’d take an eighth on my own. And eventually I started taking a quarter and with that I could feel it was ok. And when I felt I could take more, I’d add synthetic and take less brown.”*

*BV-P1*

Mixing substances appeared to be a common phenomenon which often started well before injecting drugs. The drugs which some of the heroin users interviewed injected were mostly heroin, buprenorphine and benzodiazepines, such as Rohypnol. The cocktails described during interviews are summarized in Table 3.

The main drug injected was mixed with others in order to reduce heroin consumption and satisfy cravings. In some cases, buprenorphine and street methadone were used as a way to stop heroin intake momentarily. Hence, polydrug use became a complex form of self-medication while illustrating the basic principles of economics referred to as consumer choice theory: choices made according to limited means while presented with different substitutes whose value vary according to availability and individual preferences. 

### 3.3. Perceived Benefits of Injecting Drug Use among Young People

The benefits of injecting drug use were viewed through the eyes of users. Participants often described positive side-effects and other perceived benefits of using heroin, buprenorphine or both. The types of benefits described by participants are summarized in Table 4 in terms of added value to themselves and in their relationships with others. Some of these elements were related to how well they regulated their consumption and the benefits to their personal and professional lives.

In several cases, the benefits described acted as catalysts into injecting drug use or stemmed from long-term use which comforted participants in using drugs. As the study progressed, it became clear that injecting drugs provided different forms of benefits which varied greatly according to individual experiences and beliefs.

#### 3.3.1. Value Addition to Self

In general terms, heroin was deemed to be soothing by both male and female participants. Heroin use had a better impact on one’s personal life than alcohol consumption according to the experiences of participants. Several male participants equated alcohol with violent behaviour, either in themselves or others, following the behaviour of *“relatives who act out when they are drunk, (who) ruin the parties”*. Such events acted as constant reminders of the misbehaviours associated with alcohol. Injecting drugs appeared to be a better option, both for themselves and others around them, when looking for a kick. A deeper reading of some discussions also suggests a deep-rooted form of anger among some users interviewed. This was encountered when discussing psychotropic pills, marijuana, and injecting drug use with a participant. In this specific case, marijuana was the drug of choice while injecting drug use was only an alternative. Psychotropic pills also appeared to have a similar soothing effect. Other participants were more self-aware and discussed how they could resort to violence during severe withdrawal episodes.

Several participants believed that the substances which they were injecting had special health enhancing attributes and even lifesaving properties in one case—a participant living with HIV who did not take her medication for an extended period, during what appeared to be a major depressive episode, believed that injecting heroin saved her life.

*“I was fat, as big as my sister. But it was the drugs that sustained me. Without them I think I would already have died. My CD4 had reached level 4. When that happens to people, they usually die.”*

*BC-P1*

Another participant echoed the possibility that the substances he injected had a positive effect on his health as he had not been ill for *“3 years and don’t get those things*”. He expressed the belief that he was able to fight off the flu and conjunctivitis because of heroin use. He also believed that other heroin users were able to avoid such common infectious diseases just like him. Some women interviewed described their drug use as providing a boost to their mental and physical state, allowing them to be more productive. Only one participant complained about having difficulties in working, possibly linked to her injecting what she believed might be amphetamines which she mixed with NPS.

According to several participants interviewed, another expected benefit of injecting drug use was the fact that it is cheaper than several other illicit drugs. Thus, users transitioned from other substances to heroin and other opioids, knowing that heroin was cheaper. The street price of a syrup which a participant had used previously was Rs 1500 (USD 42.8) and the price of psychotropic pills was also very high at the time of the interview. In contrast one dose (1/8 to 1/4 of heroin was worth Rs 200 to Rs 500 or USD 5.3 to 14.3) according to participants.

Traumatic personal events, such as child loss, heartbreak due to the end of a romantic relationship, and rape, were discussed in a few interviews and mentioned as acting as catalysts into either drug use or injecting drugs. The use of heroin followed shortly afterwards for two participants who lost a child during pregnancy as a way to deal with the pain of loss.

#### 3.3.2. Value Addition in Dealing with Others

Taking illicit drugs was seen by several participants as a way to bond with others or join a group. A participant started using to become more ‘street smart’ and less gullible. *“I wanted to learn how life was on the street. So that people wouldn’t be able to con me.”* Through experience, participants discovered that doses could be bought as a group and thus shared at a lowered cost, as described in some interviews. Partying and bonding with others who used drugs was also a way to overcome loneliness and family problems in other instances. Some young men mentioned that it helped them to become more confident towards women. Two interviewees reported that injecting drugs increased sexual performance. In such cases, the perceived benefits described were both deliberative and experiential. Users were encouraged by peers to see heroin as a boost before they started using and kept this outlook after a positive personal experience.

*“So a friend would walk past and say, hey, are you going to see a girl? Have something first, smoke something. You’ll be better, your girlfriend will like it. That’s how they are those youngsters. But I don’t find it to be like that. That’s what makes those young people start using. If they have to go see a girl 10 times, they’ll take drugs 10 times. And they’ve become dependent and they’ll start getting sick when they don’t have it.”*

*PL-P2*

### 3.4. Perceived Risks of Injecting Drug Use among Young Users

The fears expressed by participants concerning their drug use took many different forms. Some of these fears were experiential which included overdose or police arrests in some cases. Other perceived risks discussed by participants were learnt from peers with more experience, thus becoming deliberative risk perceptions. The fears expressed were not always plausible following inadequate knowledge (for, e.g., HIV transmission) or the root cause could not always be elaborated upon by participants, but they appeared to be affective in nature. In most cases, perceived risks encouraged heroin users to be more cautious in their drug use and injecting practices. However, these perceptions also acted as barriers to health and HR services and encouraged risky behaviours. Some of the risks attached to using illicit drugs described by participants are summarized in Table 5.

#### 3.4.1. Threats to Self

The fear of over-spending on drugs was a powerful motivator for several participants who had to find ingenious ways to both generate some form of revenue while managing their daily intake and budget. This could include having a job or finding other ways to make ends meet while leveraging on their network. Dealing drugs was however rarely seen as an option, except in one specific case where it ran in the family.

The dangers of trusting and maintaining relationships with fellow users was one of the key issues constantly mentioned by participants. Although the older, more experienced injecting drug users provided some advice and mentoring to their younger peers, the absence of trust towards peers was an aspect of injecting drug use which constantly came up during our interactions. One participant who started using during high school with her friends explained how quickly their bond faded following drug use. She believed that it was impossible to have friends when using. From her perspective, the risk of being infected on purpose by heroin users living with HIV was real: *“They’ve got it and their friends don’t, so you also need to get it. I don’t agree with that.”* She also referred to a few incidents where she saw people living with HIV sharing needles with others in the woods, knowingly, according to her. She thus kept her distance from fellow users, never shared needles and preferred to inject alone. Such experiential risks were also complemented by anecdotes from other interviews according to which fellow users could interfere with injecting equipment. Although she did not experience it personally, another participant believed that peers could easily exploit the vulnerability of users and that injecting in their presence could be lethal.

*“If someone wants to get rid of you without leaving traces, then when you’re injecting, after it’s been cooked, I just need to take one grain of salt and drop it in there. As soon as you inject, you’d die on the spot.”*

*M-P1*

Other lesser known risks linked to injecting practices include dust getting inside the needle which could lead to serious discomfort referred to as “kongolo”. Some participants mentioned this risk which appeared to be lethal in some cases:

*“And when we draw it inside the syringe, we have to use a piece of cotton wool. If the cotton wool is dirty or if there’s dust in the syringe, there’s this thing that’s called kongolo, I think you’ve heard of it? This thing that’s called kongolo, you start shaking, you’re cold and then you have a 40 C fever. Your body starts hurting just because of some dust in the needle, you start having all those complications and people can die from this.”*

*M-P1*

The participant further explained that one way of avoiding “kongolo” was to dip cotton wool in the substance in a small recipient where it had been cooked and then to place the needle through the cotton to prevent dust from getting in while injecting. She also believed that syringes should always be washed before use because dust might still be found inside, even if they were packaged and had never been used before. Several participants explained how and where they were injecting (arms, legs, genitals, behind, etc.), but few understood the risks of selecting the wrong place to inject. A participant explained that many heroin users encountered were unaware of the risk of amputation following continuous and inappropriate use of injecting equipment.

Most participants interviewed were aware of the dangers of sharing needles and the increased risk of infection: *“If the person has AIDS and he’s injected and I use the same syringe, his blood is still in there and when I inject myself, I will also get it”.* Quite a few participants reported never sharing a syringe but displayed varying levels of knowledge about different diseases: *“Yeah hepatitis is almost like HIV”.* A participant also mentioned death and hair loss as risks incurred following long-term heroin use with other substances. Most participants were aware that there were almost no external signs for people living with HIV, that infection could happen sexually and that *“it’s not written on people’s foreheads that they’ve got it. Anyone could have it without you knowing”.* While most participants were aware of voluntary counselling and testing facilities available at needle service points, and some were even tested on several occasions, others were hesitant to find out their serostatus, despite the increased risks resulting from not receiving adequate care. Such statements indicate that the risk of finding out and its potential implications were greater than not knowing.

*“That’s also how it is. That’s why with the life that I’m leading it could either be this or that. I either have it or I don’t, it’s either or. But I’d rather not know.”*

*BM-P1*

Although most participants were aware of the risk of HIV, some male participants still reported not using protection when having sexual intercourse with their wives or girlfriends. One participant initially believed that he could be infected with HIV just by using a needle, even if it had never been used before. Another participant who knew that she was HIV positive initially believed that it was acceptable to share her needle with a fellow person living with HIV. In most cases, participants were not as aware of the risks linked to hepatitis infection and its different forms.

Some users explained how they were conned and exploited by others when they first started to inject as they did not know how to use injecting equipment or where to buy drugs back then. They relied on friends, acquaintances and partners to obtain heroin and related paraphernalia and were often asked to give away their money *“But… he was only concerned with my money, he just wanted me to give him money and this became a big problem”.*

The gap in knowledge which young women had concerning illicit drugs was sometimes used against them once they were addicted. Once hooked, they were more easily convinced to engage in commercial sex work. The leverage which men had on them included how to inject and where to obtain the heroin, etc. Some participants watched their peers, friends, and even relatives start to inject and gradually becoming sex workers in order to find money to buy their dose and sometimes those of others as well. A few of these cases were shared with us.

*“Yeah, I don’t want her to do it, to have to go on the streets. Because people don’t stand on the streets because they like it. They have to do it because of their addiction.”*

*F-P2*

However, some users mentioned during interviews that they did try to preserve their partners’ dignities by preventing them from engaging in sex work.

Interviewees were aware of the risk of overdose from mixing too many substances. One participant personally overdosed on pills by taking five Tramadol pills after not feeling an immediate effect. It was only a few hours later that he fainted and then eventually woke up in the hospital. Another participant became aware of the risk of overdose following the loss of a friend. Overdose was also likely to occur when someone who had stopped using for some time then suddenly decided to inject again. This was noted among individuals who had just been released from prison or who had just exited a detoxification programme. The risk of polypharmacy was also mentioned by a participant when seeking medical assistance from a public hospital. Medical treatment was seen as potentially hazardous in the context of continuous heroin and polydrug use because of the risk of complex chemical interactions.

*“I told him (the Dr) I had taken drugs and whether it would be a problem with the injection they were giving me. if I didn’t tell him… I had to warn him.”*

*BV-P1*

Users often found themselves in situations where they knowingly adopted risky behaviours following intense cravings, despite their better judgment. This included deciding to use needles and syringes which they had previously discarded or using those of other people which they found in open areas where heroin users were known to inject. Under such circumstances users knowingly chose this option because the opportunity cost of finding a clean needle, in this case time spent feeling intense cravings, was deemed too high a cost. Another form of loss of control experienced by participants was the direct effect of living in secrecy and the resulting fear of being caught. This sometimes implied loss of control on one’s thoughts and led to paranoid behaviour where users would become suspicious of their environment.

#### 3.4.2. Threats to Others

One of the key findings of this study was that young heroin users cared about the consequences of their actions for others and not only for themselves. Disposing of syringes was a recurring theme throughout the study, with several individuals adopting different methods. In most cases, the ways in which participants disposed of syringes were motivated by the need to preserve secrecy, or the fear of additional use by others. Some also tried to minimize the impact of their injecting drug use on the community. Hence, some participants were careful and followed established protocols. Others devised their own disposal methods. For example, one participant placed his equipment in a sealed plastic bag before burying it in the ground after injecting. Another participant used construction sites to dispose of her syringes. On the other hand, injecting in the woods or in other areas without any help or supervision also encouraged users to dispose of syringes inadequately. The environmental consequences of such acts were a source of major concern which were expressed in several interviews.

Users also expressed great concern about the potential risks incurred by their family following their drug use as well as how it would affect their relationships with them. One participant who was considering having a family witnessed the impact that uncontrolled drug use had on a friend’s family. 

#### 3.4.3. Threats to Self and Others

Some analytical themes cut across the ‘self and others’ dichotomy. This included stigma and discrimination which could impact both users and their social environment. Injecting drugs was usually done alone in secret or together with fellow injectors. A recurrent theme noted throughout the study was the potential impact which injecting drug use could have on close ones. The primary concern expressed about stigma was its impact on relationships—not wanting one’s immediate family to be aware was one of the main fears expressed and was deemed to be an affective risk as emotions such as fear, worry, and shame played an important role.

*“Yeah. And… like people will talk and if your family doesn’t know you’re using and you don’t want them to know. Then you have to hide it.*

*BA-SP3*

The desire to hide drug use also extended to the parents of partners, girlfriends and wives given *“how society treats people who take drugs, they’ll say if he’s a user then he must be a thief etc.”*. The stigma experienced did not always come from close ones, or the community. It was also experienced at the community and societal levels, with multiple negative labels placed on individuals belonging to more than one affected population such as being both a druggie and an SW at the same time. Several users described society’s views about their injecting practices as being extremely negative. The stigma and discriminatory behaviour experienced by participants were also felt when trying to access healthcare. Derogatory treatment was also experienced in the private sector where patients pay for quality and discretion. These issues were only felt after users started injecting. Hence, the fears expressed by participants combined elements of both affective and experiential risks.

Pregnancy was also an issue which impacted the individual and others as explained by four young women during interviews. Two of these participants mentioned the potential health hazard of injecting drug use during pregnancy. One of them decided not to inject while the other believed she had lost a baby partly because of her drug use. She agreed to be interviewed a week after losing her child. Besides being an injecting drug user who mixed heroin with other substances, she was also HIV positive and a sex worker. She was careful during her pregnancy but had been warned by her doctor that the child would be unwell because of the illicit substances that she took. The other participant sought medical advice during her pregnancy to make sure her baby would not be ‘infected’. She stopped using heroin until after she gave birth despite being constantly pressured to use by her partner. A decrease in heroin consumption in their household meant less doses for her partner as well. She only used sleeping pills and painkillers instead during her pregnancy.

*PL-P2: “Yes, he’s the one I had a child with. It’s like he did everything to make me use again. “*

*Interviewer: “He was encouraging you to use even when you were pregnant?”*

*PL-P2: “Yeah, he tried to but I wasn’t too keen; I was scared something would happen to the baby. That he’d be disabled, that’s what I was scared of. That’s why I didn’t say anything about my drug use. I wasn’t even using. That’s all, I was scared.”*

Both participants who used heroin before being pregnant assessed the risks differently after receiving advice on how to minimize harm to the child. While one did choose to stop and the other did not, neither were aware of what to do. The risk was hence assessed experientially. The participant who chose to stop during her pregnancy had a strong emotional reaction triggered by the fear that using drugs would affect her child’s health and future. In her case, the risk was assessed affectively. The addiction of the other participant was too strong to quit.

Getting arrested by the police was a constant fear expressed by participants interviewed during the study. The risk of unwarranted arrests was thus reported as a significant obstacle to obtaining or returning clean paraphernalia from HR service points. Several participants explained that the perceived risk of being caught by the police for syringe possession limited their capacity to fully benefit from HR services, even when they were willing to take the risk to go to a caravan. Some of the implications of that fear included that several users kept using the same syringes. While in some cases the fear of being arrested was triggered by previous experiences, in most cases it came from learning or seeing what had happened to peers. A few arrests were enough to trigger constant anxiety. Young heroin users assessed problems with law enforcement deliberatively, affectively, and experientially, depending on how new they were to using heroin or connected they were to other users.

## 4. Discussion

Our study provides insights into the lived experiences of HR clients as heroin users. Our findings are consistent with economic concepts and theories which can be used to predict drug combinations and simplify the complex social environments where such practices occur. Yet, such aspects remain under-researched in Mauritius and the region. Our qualitative findings on which substances participants chose and why provides more depth to trends observed worldwide and local surveys. The analysis of the interviews revealed the subjective risk assessments participants make in relation to drug use. The cross-cutting variables identified when viewing users as consumers interacting with a wider market for drugs are (1) uncertainty and (2) reinforcing mechanisms. We thus propose a model to assess factors which influence the decision to inject drugs in Mauritius. 

### 4.1. HR Clients as Heroin Consumers

Findings from our study on drug consumption among young heroin users complement recent efforts to document drug use from competent authorities. Heroin users in Mauritius are a dynamic population as opposed to a static one. This implies that population size varies according to several contextual factors such as availability of drugs, substitutes and deaths. Some may also only inject or use heroin occasionally while others may be disappointed by the quality and availability of what is available and switch to other illicit substances. Some may even choose to stop. Despite fluctuations in quality and availability, the price of heroin appeared to stay the same unlike in other research where drug pricing was determined by social networks and cultural contexts [22]. Polydrug use among young heroin users emerged as a coping mechanism, which is well explained by consumer choice theory and triggered by the low potency of heroin. In-depth research on this form of polydrug use among young people has received limited attention in the region, but is consistent with worldwide patterns of drug use noted among the youth [23].

As in most markets, these consumers have limited information about the products available, resulting in imperfect information. A major theme that emerged in this study was the impossibility of knowing the quality of drugs being bought without trying them (only one seasoned user knew that heroin quality can be assessed by tasting). Dealers tend to know the quality of their product and sell it irrespective of its potential impact on customers with terrible results at times. This means that drug use (both for heroin and other substances) is fundamentally about trial and error until one is able to find a substance or combination that satisfies one’s needs or preferences. Drugs sold as heroin may not always turn out to be what consumers expect as per the participant who believed she was injecting amphetamines but could not prove it. This was a problem when young people mixed substances with NPS whose ingredients are unknown and often deadly. This lethal combination was believed to have caused the cases of overdose noted during that period. Illicit drug use thus opens the door to extreme levels of uncertainty which users need to find ways to cope with.

### 4.2. Uncertainty 

Our findings indicate that uncertainty is at the core of the lives of young heroin users. Users must constantly make decisions which could impact their health and affect others across most contexts [24,25,26]. They have little knowledge of the heroin market and risks involved in consuming when they first start injecting, despite starting to use other drugs such as cough syrup and pills very early in their teens. Our analysis explored how uncertainty impacted on perceived benefits and risks linked to heroin use, thus addressing some of the existing gaps in the literature.

Users appear to navigate the risks and benefits of drug use informed by existing, non-existing, or inadequate information and peer influence. Perceived benefits of injecting drug use differed greatly across individuals. Most benefits described by participants were a mix of affective and deliberative and were hence deemed to be experiential, with the exception of lower monetary costs of injecting drugs compared to using other illicit substances. Both knowledge and circumstances linked to drug use also varied greatly among users. Despite such variance across individuals, the perceived benefits associated with illicit drug use were strongly associated with the underlying factors leading to the initiation into smoking or injecting heroin such as peer influence, access to drugs or intimate relationships. These perceived benefits gradually became experiential over time. The most striking example in this category is enhanced sexual performance for young men which also suggests the existence of a ‘chemsex’ culture in Mauritius.

We also found that injecting practices are influenced by both deliberative perceived risks (e.g., not sharing needles) and experiential risks (e.g., deciding not to inject with peers as they cannot be trusted). Affective risks play a more significant role in shaping and encouraging risky behaviours. Stigma is quite prevalent among heroin users and people living with HIV in Mauritius [27]. Awareness of the direct and indirect negative impact of stigma and discrimination on close ones increases the need for secrecy. This encourages heroin users to adopt coping strategies such as using dirty needles and decreases their willingness to reach out to services and ask for peer support. The fear of arbitrary arrests by the police, or of being exposed to close ones, often encourages heroin users to adopt a double life and inject in the woods or in secluded areas. Although injecting in the woods increases privacy and reduces the chances of public exposure, it also encourages users to adopt risky injecting practices, which significantly increase the risk of HIV, hepatitis infection as well as overdose without anyone nearby to intervene. This also leads to needles and syringes being hidden in remote locations, which also increases the risk that others in desperate need of injecting equipment find and use the items stored or not discarded properly.

### 4.3. Reinforcing Mechanisms

The reinforcing mechanisms documented in the context of drug use include those linked to addiction which focus on physiological and clinical effects as well as those promoting abstinence at the community level [28,29]. Our results point to the existence of both internal and external reinforcing mechanisms which encouraged heroin use by promoting the positive effects of the substance. The main pre-requisite for framing drug injection as acceptable and beneficial is the presence of acquaintances, partners, friends, or family who are either injecting drugs or are connected to drug users. This factor acts as a reinforcing mechanism in most cases, suggesting that bonding with others through injecting drug use also shapes how positively users viewed their own personal heroin consumption. The fear of being HIV positive is an example of an internal emotional and psychological burden which acts as a major barrier for young people who might consider to stop using drugs. A fatalist attitude settled in whereby young people continue to use substances as a means of escape without being able to seek help or confide in their relatives or close ones because of stigma. This self-imposed inability to open up may discourage individuals from having meaningful or committed relationships (even one participant explained that he would never have participated in the study if he had been HIV positive). Addiction and cravings are described by participants as internal reinforcing mechanisms for unsafe injecting practices rather than drug use itself. Such examples further highlight the need for effective and accessible HR services for young people where informal service providers such as hit doctors can play a more significant role as recently shown in the emerging literature [30].

### 4.4. Towards a Cohesive Model for Injecting Drug Use in Mauritius

**Figure 2:**
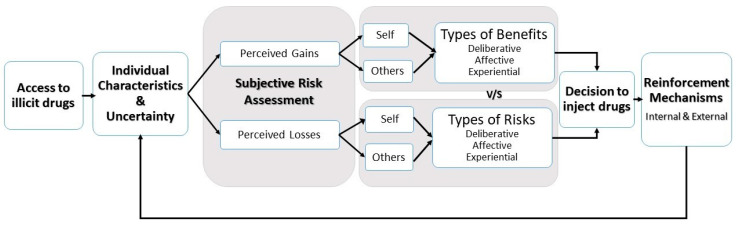
Factors actors influencing the decision to inject drugs in the Mauritian context.

Our interactions with young heroin users show that economic models and theories can simplify the dynamics of injecting drug use in the Mauritian context. We gradually expanded our initial framework of perceived risks and benefits shown in Section 1.2 to include the perceived impact on others as well as uncertainty and reinforcing mechanisms. ‘Access to illicit drugs’ is complemented by ‘individual circumstances’ to acknowledge that the decision to use drugs can include factors such as intimate relationships, personal well-being, drug history, etc. Not all individuals who can access drugs automatically start using, as explained by participants. Reinforcing mechanisms are likely to have a higher influence and encourage continuous use among individuals with specific characteristics and who are not knowledgeable about heroin to begin with. A summary of our proposed model and the key variables identified are illustrated in Figure 2.

The implications of this model for preventing injecting drug use and health consequences among those who are already injecting are quite straightforward. We expect that tackling uncertainty and reinforcing mechanisms by promoting awareness of real-life dangers will have a positive impact on the lives of young people using drugs. It would also be highly effective to counsel young people who are only experimenting with pills, NPS, and other substances about the risks linked to injecting drug use and to expose the falseness of the perceived benefits which they are likely to hear from peers. For those who are already injecting, it is extremely important to learn about the true dangers linked to risky injecting practices and reinforcing mechanisms potentially fuelled by their initial lack of experience.

## 5. Limitations and Reflections

Key affected populations by HIV and hepatitis such as MSM and transgender individuals could not be identified within this age-group. It is also possible that other types of heroin users who also injected other substances besides those enumerated exist outside of the sample of participants, with additional combinations of illicit drugs resulting in additional risks than those reported in this sample. Several delays in identifying and recruiting participants were experienced following the absence of a functional network of people who use drugs, which could have eased the recruitment process and provided additional advice. We also note that some accounts of the benefits linked to injecting heroin were counter-intuitive, especially those in relation to sexuality and perceived health benefits. It is also likely that other hidden risks were not unveiled following the small size of the sample, which may be revealed in future studies. The contribution of service providers to themes tackled in this study was limited but will be given more weight during a secondary analysis of the data focusing on access and barriers to HR services.

## 6. Conclusions

The changing nature of the risks perceived by young heroin users illustrates the need to build upon HR interventions made possible by the local HIV/AIDS Act of 2006 to develop a new generation of HR strategies specific to the context. Such strategies could factor in risk stratification and risk communication specific for young heroin users, couples, and their communities but would need to be informed by additional research. New types of service providers such as hit doctors and volunteers could be empowered. Testing of substances to inform consumers could also be undertaken with the support of the scientific community. Exploring the type of risks and benefits perceived by young heroin users can help service providers to better understand the incentives to which their clients respond. Such information could be used to develop services targeted towards the youth to prevent the transmission of HIV, HBV and HCV, as well as other potential threats from uncontrolled drug use and injecting practices.

There is a strong evidence-base for most HR services in Mauritius, but it is crucial to adopt a more participatory and inductive approach to programme monitoring and surveys carried out. Our study shows that qualitative research which explores contextual rationality can yield in-depth socio-behavioural insights and potentially help to design more responsive surveys. Several sensitive issues such as drug use and sexuality, exploitation, and control of young women would have been overlooked by a conventional quantitative exercise. Such themes need to be investigated with the help of a functional network of drug users. The unique expertise of users would also help hidden sub-groups whose needs are unknown to emerge.

Our findings also suggest that the economics of injecting drug use are under-researched in Mauritius. Previous surveys carried out on the perceptions of drugs in the general population could be adapted and administered to heroin users themselves. Complementary information on out-of-pocket expenses, knowledge, attitude, and the quality of information available about injecting practices could be more useful for policy makers than information on self-reported behaviours, especially among young heroin users. Carrying regular cost benefit analyses can also help to compare the societal costs of existing HR services to drug control policies with a view to guide investment. However, the findings of such exercises may potentially differ if outcomes are stratified according to age-group and if the costs of deaths, amputations, accidents and life-long chronic conditions such as HIV and hepatitis are taken into consideration.

## Figures and Tables

**Table 1:** Participant categories, N = 27.

Total	People Who Used Drugs	Service Providers/ Peer Educator
Of whom		
Men	11	3
Women	11	2
Currently use Heroin (Inject/Smoke)	19	1
Injected Heroin over the past 4 months	3	1
Enrolled on Methadone but injected in the past 2 months	1	
On codeine but injects occasionally	1	
Combined Heroin with other substances	7	2
Injected other substances (buprenorphine, Rivotril, amphetamines)	2 (1 injected both Rivotril and buprenorphine)	1
Are HIV positive (disclosed during interview)	2	1
Are HCV positive	Undisclosed	Undisclosed

**Table 2:** Street price of illicit drugs.

Drug	Street Price (Rs)	US Dollars 1 USD = Rs 35 (2017)
Heroin	1/8 g at 400	11.5
	1 g at 4500	128.5
New Psychoactive Substances	100	2.85
Cough Syrup	Kaffan 150	4.3–7.5
	Benylin 60	1.7
	Benecot 700	20
	Phensezyl 1000	28.6
	Others may go up to 1500	43
Rivotril	100	2.85
Marijuana	2000	57.1
Methadone	250–2000 (depending on quantity)	57.14
Nova, Zamidol, Dramal, Dramazac, Rohypnol	40–60 a pill, tablets at 400–600	11.4–17.1

**Table 3:** Polydrug use among heroin users.

Illicit Drug Injected	Additional Substances Mentioned	
	Complements	Used as Regulator/to Stop Momentarily
Heroin	Cough Syrup/Codeine	
	Analgesics (Tramal)	Street methadone
	Tranquilizers	
	New Psychoactive Substance (Sintetik)	Buprenorphine
	Marijuana	
	HIV medication	Codeine
	Alcohol	
Buprenorphine	Tranquilizer	Codeine
	Cough Syrup/Codeine	
	Analgesics	
	Valium	
	Rivotril	
Benzodiazepines or Rohypnol	Used with Heroin and others	Buprenorphine
Amphetamines (possibly mixed with other unknown substances)	New Psychoactive Substance	Street methadone

**Table 4:** Perceived benefits of Heroin use among heroin users.

Major Theme	Analytical Theme	Code	Type of Perceived Benefit	Illustrative Quotes
**Perceived Benefits of Injecting Drug Use**	Value addition to self	Mood enhancement and increased well-being	Experiential	*“Drugs help me express myself. And yet, when I use, it helps me to not think, almost like it allows me to relax.”* *BC-P1*
		Better health		*“I’ll be frank, ever since I started using, I haven’t been ill. I don’t have fevers, flus; I don’t get any of those.”* *BA-P2*
		Increased productivity		*“But in general when I was using, it gave me more strength to work, I could do twice the amount of work I usually do.”* *M-P1*
		Lower costs of injecting drug use	Deliberative	*“But… they’re too expensive. I didn’t… how to say it… I didn’t have enough money to continue with the pills so I started taking drugs” * *BV-P5*
		Dealing with trauma	Experiential	*“Err… yeah. In a way, yes, when I saw them like that, I thought that my suffering could be allayed with that. That’s what made me do it”* *M-P1*
	Value addition in dealing with others	Bonding and experimenting	Experiential	*“It’s through the psychotropic pills that I started needing it. When we went to birthday parties, I would see loads of it. And friends would say, come let’s smoke, let’s smoke… I don’t drink, you understand, we would sit, ten of us and we’d all smoke, you understand. And there were lots of it… that’s how I developed the craving.”* *F-P2*
		Intimate relationships and sexuality	Deliberative and experiential	*“And like, once I got used to it, it was different. It’s like it was better, you understand? When I talked to her, there were lines that came out of my mouth, I could talk, I was on a cloud.”* *BV-P3*

**Table 5:** Perceived risks of illicit drug use among heroin users.

Major Theme	Analytical Theme	Code	Type of Perceived Risk	Illustrative Quotes
**Perceived Risks of Injecting Drug Use**	Threat to self	Escalating Costs	Experiential	*“Yes, it’s more expensive. An eighth costs Rs 500 (USD 14.2) If I take two of those, that’s Rs 1000 (USD 28.5). If I have to take two more, that’s Rs 2000 (USD 57.0)”* *BV-P1*
		Peers		*“No well… you can’t… to be honest, you can’t… those kinds of people… you can’t. And this also applies to me, you can’t consider people like that as friends”* *F-P1*
		Injecting practices		*“Yeah, sometimes. Where it’s better to inject, like look here in my hand, those veins that look green? Like here? You’re not supposed to inject here, that’s risky. No green veins because they’ll swell, it’ll do…it’s not good.”* *M-P1*
		Side-effects and infectious disease		*“If the person has AIDS and he’s injected and I use the same syringe, his blood is still in there and when I inject myself, I will also get it”.* *PL-P3*
		Exploitation		*“I later found out that I wasn’t the only one he encouraged to inject and then once they were hooked… he did it before with another girl. He had already…. selling the girl… “* *F-P3*
		Overdose and polydrug use	Deliberative	*“He was inside, sitting and he had already taken pills, he’d had cough syrup, he’d taken a lot of it and was having a good high. Then he injected an eighth of brown and it was too strong. He couldn’t take it.”* *BV-P1*
		Loss of control	Experiential/ affective/ deliberative	*“Well if I do it, I think about the problems I could get. Diseases and what not. But like I said to you, this drug is more powerful than anything else.”* *F-P1*
	Threat to others	Disposal of syringes	Deliberative	*“I prefer to burn it because if you dispose of it in a trashcan, children could find it and hurt themselves.”* *BC-P1*
		Couple/family	Affective	*“And then I realised that I couldn’t love drugs more than I love my children, more than I loved the family that I have built.”* *M-P1*
	Threat to self and others	Stigma and discrimination	Affective/ experiential	*“Because when society looks at us, they don’t approve. We can’t find work. Especially if we engage in immoral activities. It’s hard for us.”* *BC-P1*
		Pregnancy	Deliberative/ affective/ experiential	*“Yes, I feel ok. The doctor told me the child could be sick. And despite all the precautions I’ve taken, my belly was small. It was small like that (shows belly…) because of the drugs.”* *BC-P1*
		Law enforcement	Deliberative/ affective/ experiential	*“If I go to the caravan to take some syringes and I take them home. What if as I’m taking the old ones back, the police arrest me? That’s a problem as they could charge me.”* *F-P1*

## References

[1] Ministry of Health and Quality of Life Health Statistics Report 2017. http://health.govmu.org/English/Statistics/Health/Mauritius/Pages/default.aspx.

[2] Pathack A., Saumtally A., Kinoo S.A.H., Comins C., Emmanuel F. (2018). Programmatic mapping to determine the size and dynamics of sex work and injecting drug use in Mauritius. Afr. J. Aids Res..

[3] Ministry of Health and Quality of Life (2016). National Drug Observatory Report.

[4] Prevention Information et Lutte contre le Sida/Talor Nelson Sofres Image and Perceptions of Drugs in Mauritius. http://pils.mu/wp-content/uploads/2017/03/TNS-Image-and-perception-of-drugs-in-Mauritius.pdf.

[5] Ministry of Health and Quality of Life, National AIDS Secretariat Integrated Biological and Behavioural Surveillance Survey among People Who Inject Drugs 2017: A Respondent Driven Survey (RDS) among People Who Inject Drugs [PWIDs] in the Island of Mauritius. http://cut.mu/wp-content/uploads/2018/12/IBBS-Survey-report-for-PWIDs-2017.pdf.

[6] United Nations Office on Drugs and Crime World Drug Report 2010 Annex 8.4 Price and Purity. www.unodc.org›field›8.4_Price_Purity_Opioids.xlsx.

[7] United Nations Office on Drugs and Crime World Drug Report 2005, Volume 2, Section 7.1: Opiates: Wholesale, Street Prices and Purity Levels. https://www.unodc.org/pdf/WDR_2005/volume_2_chap7_opiates.pdf.

[8] Ministry of Health and Quality of Life 2nd National Drug Observatory Report. http://health.govmu.org/English/Documents/2018/NDO_MOH_FINAL_JOSE_VERSION_05July_2018Brown.pdf.

[9] UNAIDS 2018 Country Data for Mauritius. https://www.unaids.org/en/regionscountries/countries/mauritius.

[10] Ministry of Health & Quality of Life HIV/AIDS Statistics. http://health.govmu.org/English/Documents/2018/HIVDec%202017.pdf.

[11] Collectif Urgence Toxida (2017). Internal Monitoring & Evaluation Reports.

[12] Ministry of Health & Quality of Life (2017). Internal Monitoring & Evaluation Reports.

[13] Kahneman D., Tversky A. (1979). Prospect Theory: An Analysis of Decision Under Risk. Econometrica.

[14] Caulkins J.P., Kleiman M.A.R., Tony M. (2011). Drugs and Crime. The Oxford Handbook of Crime and Criminal Justice.

[15] Piore M.J. (2006). Qualitative research: Does it fit in economics?. Eur. Manag. Rev..

[16] Ferrer R., Klein M.W. (2015). Risk Perception and Health Behaviour. Curr. Opin. Psychol..

[17] Rhodes T. (1997). Risk Theory in Epidemic Times: Sex, Drugs and the Social Organisation of ‘Risk Behaviour’. Sociol. Health Illn..

[18] Rhodes T., Wagner K., Strathdee S.A., Shannon K., Davidson P., Bourgois P. (2011). Structural Violence and Structural Vulnerability Within the Risk Environment: Theoretical and Methodological Perspectives for a Social Epidemiology of HIV Risk Among Injection Drug Users and Sex Workers. Rethinking Social Epidemiology.

[19] Goudie A.J., Sumnall H.R., Fild M., Clayton H., Cole J.C. (2007). The effects of price and perceived quality on the behavioural economics of alcohol, amphetamine, cannabis, cocaine, and ecstasy purchases. Drug Alcohol Depend..

[20] Creswell J.W. (1998). Qualitative Inquiry and Research Design: Choosing among Five Traditions.

[21] Glaser B.G., Strauss A.L. (1967). The Discovery of Grounded Theory: Strategies for Qualitative Research.

[22] Moller K., Sandberg S. (2019). Putting a price on drugs: An economic sociological study of price formation in illegal drug markets. Criminology.

[23] United Nations Office on Drugs and Crime World Drug Report 2018: Drugs and Age Drugs and Associated Issues among Young People and Older People. https://www.unodc.org/wdr2018/prelaunch/WDR18_Booklet_4_YOUTH.pdf.

[24] Mital S., Miles G., McLellan-Lemal E., Muthui M., Needle R. (2016). Heroin Shortage in Coastal Kenya: A Rapid Assessment and Qualitative Analysis of Heroin Users’ Experiences. Int. J. Drug Policy.

[25] Garami J., Haber P., Myers C.E., Allen M.T., Misiak B., Frydecka D., Moustafa A.A. (2017). Intolerance of Uncertainty in Opioid Dependency—Relationship With Trait Anxiety and Impulsivity. PLoS ONE.

[26] Ciccarone D., Ondocsin J., Mars S.G. (2017). Heroin Uncertainties: Exploring Users’ Perceptions of Fentanyl-Adulterated and Substituted ‘Heroin’. Int. J. Drug Policy.

[27] Prevention Information et Lutte Contre le Sida The People Living with HIV Stigma Index. http://pils.mu/wp-content/uploads/2018/10/STIGMA_F.pdf.

[28] Wise R.A., Foob G.G. (2014). The Development and Maintenance of Drug Addiction. Neuropsychopharmacology.

[29] Roozenab H.G., Boulognea J.J., van Tulderc M.V., van den Brink W., De Jong C.A.J., MKerkhofa J.F. (2004). A systematic review of the effectiveness of the community reinforcement approach in alcohol, cocaine and opioid addiction. Drug Alcohol Depend..

[30] Stengel C.M., Marie F., Guise A., Pouye M., Sigrist M., Rhodes T. (2018). They accept me, because I was one of them: Formative qualitative research supporting the feasibility of peer-led outreach for people who use drugs in Dakar, Senegal. Harm Reduct. J..

